# EGF activates TTP expression by activation of ELK-1 and EGR-1 transcription factors

**DOI:** 10.1186/1471-2199-13-8

**Published:** 2012-03-20

**Authors:** Magdalena Florkowska, Piotr Tymoszuk, Aleksandra Balwierz, Anna Skucha, Jakub Kochan, Mateusz Wawro, Krystyna Stalinska, Aneta Kasza

**Affiliations:** 1 Deptartment of Cell Biochemistry, Jagiellonian University, Gronostajowa, Poland; 2 Department of Cell Biochemistry, Faculty of Biochemistry, Biophysics and Biotechnology, Jagiellonian University, Gronostajowa 7, 30-387, Kraków, Poland

## Abstract

**Background:**

Tristetraprolin (TTP) is a key mediator of processes such as inflammation resolution, the inhibition of autoimmunity and in cancer. It carries out this role by the binding and degradation of mRNA transcripts, thereby decreasing their half-life. Transcripts modulated by TTP encode proteins such as cytokines, pro-inflammatory agents and immediate-early response proteins. TTP can also modulate neoplastic phenotypes in many cancers. TTP is induced and functionally regulated by a spectrum of both pro- and anti-inflammatory cytokines, mitogens and drugs in a MAPK-dependent manner. So far the contribution of p38 MAPK to the regulation of TTP expression and function has been best described.

**Results:**

Our results demonstrate the induction of the gene coding TTP (*ZFP36*) by EGF through the ERK1/2-dependent pathway and implicates the transcription factor ELK-1 in this process. We show that ELK-1 regulates *ZFP36 *expression by two mechanisms: by binding the *ZFP36 *promoter directly through ETS-binding site (+ 883 to +905 bp) and by inducing expression of EGR-1, which in turn increases *ZFP36 *expression through sequences located between -111 and -103 bp.

**Conclusions:**

EGF activates TTP expression via ELK-1 and EGR-1 transcription factors.

## Background

Gene *ZFP36 *encodes for tristetraprolin (TTP, also known as G0S24, ZFP36, TIS11, and Nup475). The gene product is the prototype of the tandem CCCH zinc finger protein family, called TIS11, which includes four structurally and sequentially related proteins - TTP, BRF-1, BRF-2 and ZFP36L3 [1]. TIS11 family members feature a conserved non-typical tandem zinc finger domain that mediates its interaction with target RNA species [2]. TTP is the most thoroughly described member of the TIS11 family, and has been identified as a nucleo-cytoplasmic protein that specifically binds mRNAs containing Adenine/Uridine-Rich Elements (AREs) in their 3'-UTRs (3'-untranslated regions) and directs them to exosome- or P-body-mediated degradation [3]. Genome-wide experiments have revealed many potential targets for TTP-mediated degradation. A more direct approach confirmed that TTP interacts with transcripts encoding for a number of cytokines (TNFα, IL-1β, IL-2, IL-6, GM-CSF), pro-inflammatory factors (iNOS, COX-2), proteins which have important roles in breast cancer invasion and metastasis (urokinase, urokinase receptor, metalloproteinase-1, VEGF), immediate-early response proteins like c-FOS and tristetraprolin itself [4-6].

The activity of TTP is regulated predominantly via its phosphorylation by MAP kinases (in particular p38 and ERK1/2), kinases downstream of MAPKs (including MK2, the p38 substrate or MK3) or AKT in response to pro-inflammatory cytokines (TNFα, IL-1β, IFNγ), LPS and anti-inflammatory factors (TGFβ, dexamethasone). The TTP protein sequence contains several key serine/threonine residues, the modification of which was previously shown to determine the ability of TTP to recruit the mRNA-degradation machinery, bind transcripts, be shuttled to P-bodies or stress granules and its nuclear/cytoplasmic localization and protein stability [7-12].

Less is known about the regulation of TTP expression. Both mRNA and protein are induced rapidly after stimulation of the cells with pro- and anti-inflammatory factors (TNFα, LPS, IL-1β, IL-4, TGFβ, IFNγ, glucocorticoids as well as mitogenic factors such as those found in serum, or phorbol esters) [11,13-18]. The structure of the murine *Zfp36 *gene promoter has been characterized, with the identification of a conserved proximal EGR-1 transcription factor binding site, AP2, SP1, TTP promoter element 1, STAT6 and SMAD biding sites and a functional GAS element. The first intron has also been shown to play a key role in mitogen-induced expression of *Zfp36 *[18,19].

The ELK-1 transcription factor is a representative member of ETS protein family characterized by the presence of the evolutionary conserved ETS domain stabilized by three key tryptophan residues and responsible for the interaction with DNA [20]. The ELK-1 domain structure includes an ETS domain (known also as Box A) at the N-terminus, Box B domain in the middle part of the sequence and C-terminal transactivation domain (TAD, Box C). Box B is also found in other members of TCF (Ternary Complex Factor) subfamily (SAP-1, SAP-2) and is relevant for the formation of ternary complex with SRF (Serum Responsive Factor) on SREs (Serum Response Element) [21]. Box A is a site of recruitment of the mSIN3A/HDAC1 complex, which confers the repressor function of ELK-1 [22]. HDAC-2 is recruited to ELK-1 through the repressive R-motif in SUMO-dependent manner. The phosphorylation of the TAD serine/threonine residues is crucial for switching from repression to activation of transcription [23]. The phosphorylation is catalyzed mainly by MAPKs such as ERK1/2 and Ser383 phosphorylation serves as a hallmark of ELK-1 activation. Only a handful of the genes directly targeted by ELK-1 are known. Among them *EGR-1 *and *FOS *seem to have the most important function in the regulation of the immediate-early cell response and widen the spectrum of ELK-1-regulated genes [24,25]. The role of ELK-1 in the regulation of immunological response has also been emphasized [26].

## Results

### EGF regulates tristetraprolin expression in ERK1/2-dependent manner

**Figure 1:**
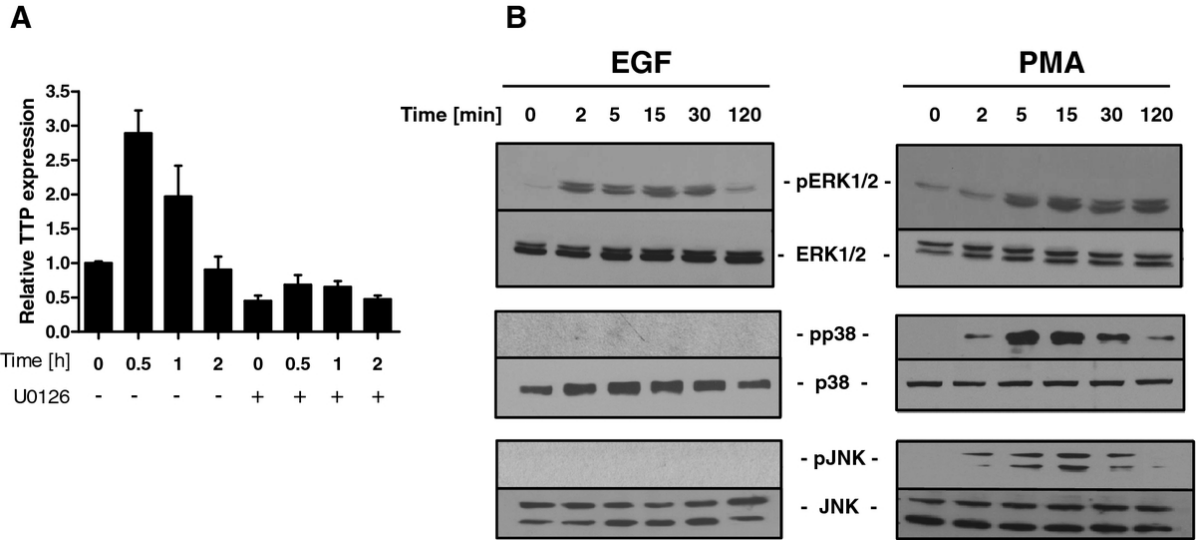
**(A) TTP mRNA expression upon EGF stimulation**. Serum-starved MCF-7 cells were stimulated with EGF (20 ng/ml) for indicated times. The total RNA was extracted and analyzed by Real Time PCR using *ZFP36 *specific primers. **(B) Activation of ERK1/2 pathway upon EGF stimulation**. Serum-starved MCF-7 cells were stimulated with EGF (20 ng/ml) or PMA (100 ng/ml) for indicated times, harvested and subjected to Western blot analysis (with anti-phospho ERK1/2, anti-ERK1/2, anti-phospho p38, anti-p38, anti-phospho JNK and anti-JNK antibodies)

Stimulation of the human breast cancer MCF-7 cell line with EGF resulted in a rapid induction of TTP expression, the maximal effect being observed 30 minutes after EGF treatment. The ERK1/2 pathway inhibitor, U0126, inhibited this process (Figure 1A). In order to confirm the involvement of EGF in the activation of ERK1/2 in the MCF-7 cell line we performed western blot analysis using anti-phospho ERK1/2, anti-phospho p38 and anti-phospho JNK antibodies. We found that EGF was a specific activator of ERK1/2 phosphorylation in this system (Figure 1B). We were unable to detect the phosphorylation of JNK or p38 after EGF treatment whereas PMA treatment induced phosphorylation of all tested MAP kinases (Figure 1B). We concluded that EGF activates the ERK1/2 pathway in the MCF-7 cell line and that activation of this pathway resulted in increase of TTP mRNA.

### Regulation of *ZFP36 *promoter by EGF

**Figure 2:**
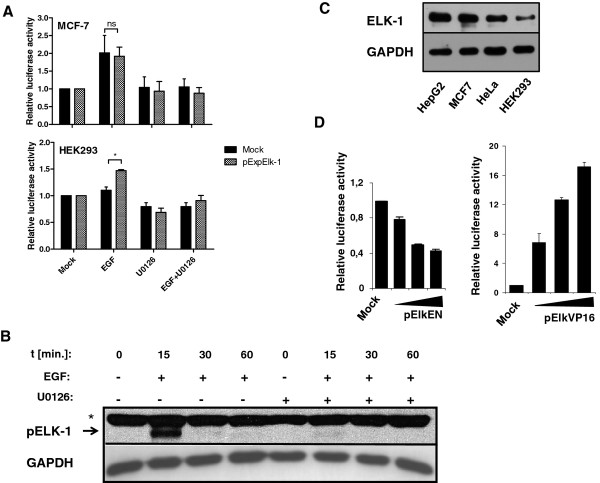
**Activation of *ZFP36 *promoter upon EGF stimulation**. **(A) **MCF-7 or HEK293 cells were transfected with reporter vector containing full-length *ZFP36 *promoter fragment (-488 to +905 bp) and, where indicated, with ELK-1 expression vector (pExpELK-1). 24 hrs after transfection cells were stimulated for 8 hrs with EGF with or without U0126 pretreatment and then luciferase activity was measured. **(B) **Serum-starved MCF-7 cells were stimulated with EGF (20 ng/ml) for indicated times, harvested and subjected to Western blot analysis (with anti-phospho ELK-1 and anti-GAPDH antibodies as loading control). Asterisk indicates unspecific band **(C) **The total cellular proteins from different cell lines were isolated and subjected to Western blot analysis (with anti-ELK-1 antibody and anti-GAPDH antibodies as loading control). **(D) **MCF-7 cells were co-transfected with reporter vector containing full-length *ZFP36 *promoter fragment (-488 to +905 bp) and indicated amounts of pElk-EN or pElk-VP16. 24 hrs after transfection luciferase activity was measured. **(A and D) **Representative results from three independent experiments are shown and plotted as means ± SD (n = 3)

We decided to test the hypothesis that ERK-mediated expression of TTP is regulated at the promoter level. We therefore generated a reporter construct containing the *ZFP36 *promoter fragment (-488 to +905 bp), further abbreviated as ZFP36. The promoter fragment included the first exon, intron and the upstream promoter sequence of *ZFP36 *gene. This promoter fragment was activated after EGF treatment, and this activation was blocked by the ERK1/2 pathway inhibitor, U0126 (Figure 2A). ELK-1, a well-characterized substrate of ERK1/2, is phosphorylated on Ser383 after ERK1/2 activation. We therefore investigated ELK-1 Ser383 phosphorylation status upon EGF treatment, and observed an increase after 15 minutes of EGF stimulation (Figure 2B). As all canonical MAPK are capable of phosphorylating ELK-1 on Ser383, we found that inhibition of ERK1/2 using U0126 abrogated the observed phosphorylation. We also confirmed the expression of ELK-1 in MCF-7 cells at the protein level, and found that ELK-1 level was relatively high in comparison to other tested cell lines (Figure 2C).

Having shown that ELK-1 is activated by ERK1/2 in response to EGF stimulation, we checked whether the promoter of *ZFP36 *is the target of ELK-1 regulation. For this purpose we used the ZFP36 reporter vector and expression vectors for constitutively active and dominant-negative forms of ELK-1, Elk-VP16 and Elk-EN, respectively. Due to the presence of strong and non-facultative activation (VP16) or repression (EN) domains fused in frame with C-terminus of ELK-1, the transcription factor acts independently of MAPK activation while preserving the DNA binding specificity [27]. ZFP36 is activated by Elk-VP16 and repressed by Elk-EN in a dose-dependent manner (Figure 2D). The intensity of activation of the promoter by Elk-VP16 is much stronger than the intensity of activation observed after EGF treatment. One possible explanation of this phenomenon may be that VP16 domain conjugated to ELK-1 is more powerful on *ZFP36 *promoter than phosphorylated form of ELK-1. Dose-dependent regulation of activation/repression of *ZFP36 *promoter by Elk-VP16 or Elk-EN suggests that observed regulation is specific.

In the mock-transfected MCF-7 cells, EGF stimulation caused an increase in promoter activity, which was completely abolished by the inhibition of ERK1/2 (Figure 2A). The transient overexpression of native ELK-1 did not change the fold of stimulation by EGF in MCF-7 cell line, which already expresses high levels of endogenous ELK-1 (Figure 2C). In HEK293 cell line with very low level of ELK-1 (Figure 2C) the overexpression of this transcription factor restored the responsiveness of *ZFP36 *promoter to EGF. In MCF-7 as well as ELK-1 transfected HEK293 cells the EGF-dependent activation of the promoter was blocked by U0126. This made us conclude, that ELK-1 can be engaged in the regulation of *ZFP36 *promoter after EGF stimulation. These findings were further supported by the results obtained by qPCR. In ELK-1-limited HEK293 cells the stimulation of TTP expression by EGF is not observed (data not shown).

### Sequences in *ZFP36 *promoter responsible for ELK-1 regulation

**Figure 3:**
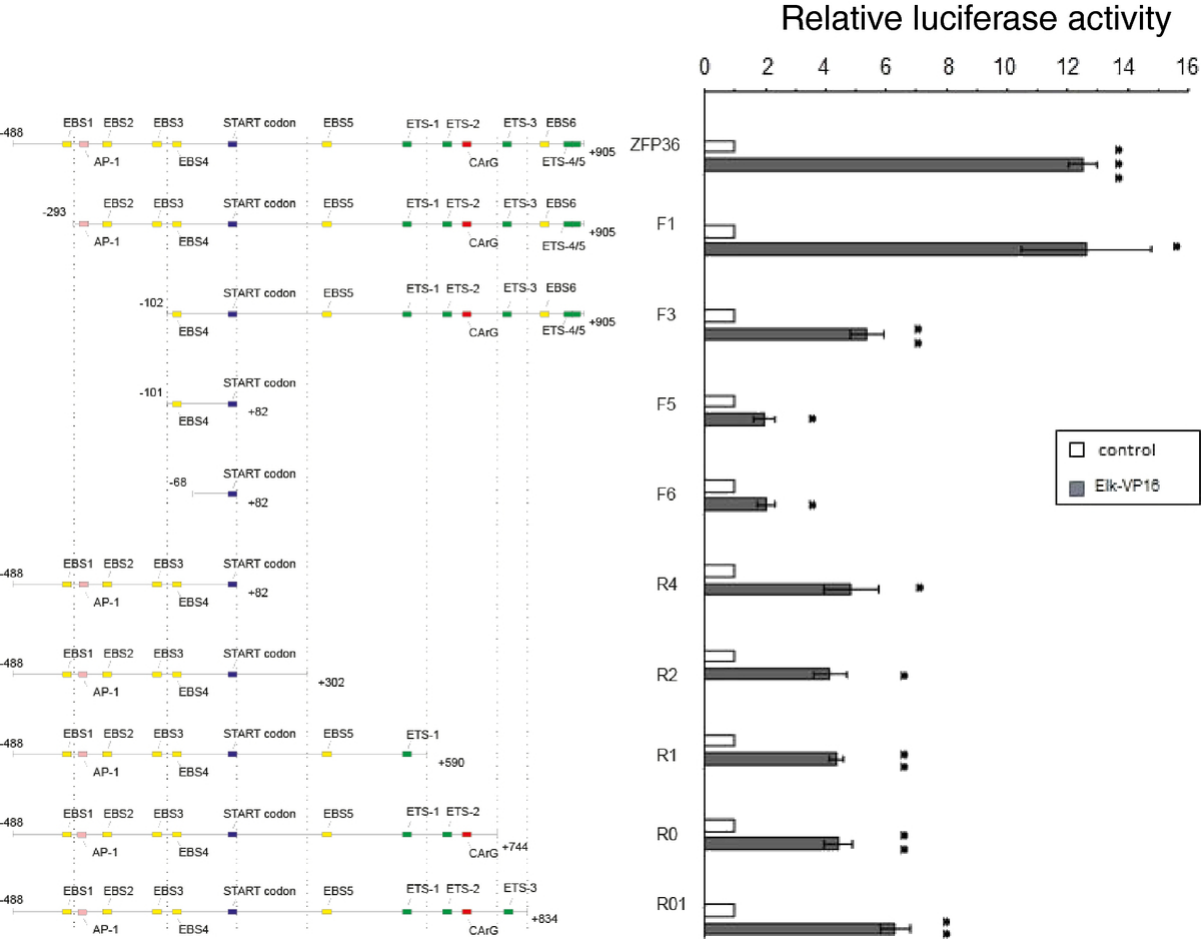
**Activation of *ZFP36 *promoter by Elk-VP16**. A series of deletion mutants of *ZFP36 *promoter was cloned and co-transfected into MCF-7 cells with/without 50 ng pElkVP16. 24 hrs after transfection cells were harvested and luciferase activity was measured. Results from three independent experiments are normalized to the mock-transfected control (without pElk-VP16) and plotted as means ± SD (n = 3), * *P *< 0,05, ** *P *< 0,01, ***P < 0,001

In order to find sequences involved in the observed regulation of *ZFP36 *promoter by ELK-1 we designed a series of truncation mutants. Deletion of two regions, -293 to -103 bp and +744 to +905 bp, has resulted in a substantial decrease in promoter activation by Elk-VP16 (Figure 3).

**Figure 4:**
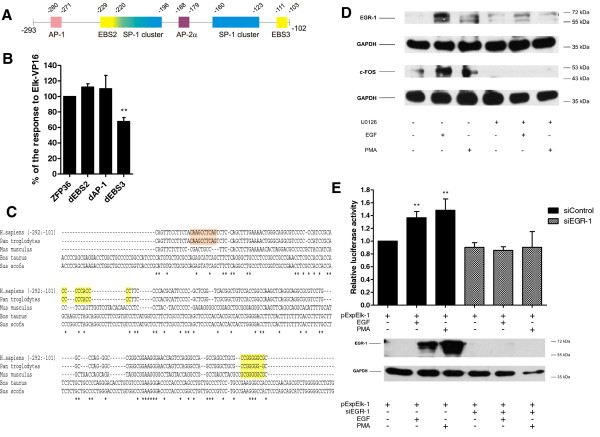
**Analysis of importance of sequences present in -293 to -102 bp *ZFP36 *promoter fragment**. **(A) **The potential transcription factor binding sites, identified in analyzed promoter fragment by Alibaba 2.1 and TRANSFAC 6.0 programs, are shown. **(B) **MCF-7 cells were co-transfected with full-length wild type *ZFP36 *promoter or with mutation in EBS2 (dEBS2), AP-1 (dAP-1) or EBS3 (dEBS3) and 50 ng of pElk-VP16. 24 hrs after transfection luciferase activity was measured. Results are shown as a percentage of activation of mutated variant of the promoter in comparison to the full length -488 to +905 bp fragment, both stimulated with Elk-VP16. Data are presented as mean value ± SD from three independent experiments (**P < 0,01) **(C) **-292 to -102 bp fragments of *ZFP36 *promoter originating from different species were analyzed using the Emboss suite of programs. Sequence homologues were found by direct search across EMBL database and pairwase alignment with Smith-Waterman algorithm, using standard parameters (GOP = 10, GEP = 1). Figure shows results of multiple alignment performed with ClustalW2 program (GOP = 10, GEP = 5) and the color-highlighted fragments point the transcription factors binding sites, identified by TESS program working on TRANSFAC6 database, and potentially important for the activation of *ZFP36 *promoter by Elk-VP16 **D) **Serum-starved MCF-7 cells were stimulated with EGF (20 ng/ml) or PMA (100 ng/ml) for 2 hrs with or without U0126 pretreatment. Then the cells were harvested and subjected to the Western blot analysis with anti-EGR-1, anti-c-FOS and anti-GAPDH antibodies. **(E) **The MCF-7 cells were co-transfected with ZFP36 and pExpELK-1 and either EGR-1 targeted siRNA or control siRNA. 18 hrs after transfection serum-starved cells were stimulated with EGF (20 ng/ml) or PMA (100 ng/ml) for 8 hrs. The relative luciferase activity was measured. The bottom panel shows the results of Western blot analysis with anti-EGR or anti-GAPDH antibodies in the lysates collectted from serum-starved MCF-7 cells transfected with EGR-1 targeted siRNA or control siRNA and stimulated 2 hrs with EGF or PMA.

The region -293 to -103 bp contains two EBS sites (EGR-1 binding sites), namely EBS2 and EBS3 and one AP-1 binding site (Figure 4A). We introduced point-mutations into each of these sites (dEBS2, dEBS3 or dAP-1) in the full-length *ZFP36 *promoter. Results of these experiments exclude the role of AP-1 and EBS2 binding sites in the investigated regulation (Figure 4B), despite the fact that ELK-1 can stimulate the expression of both c-FOS (AP-1 component) and EGR-1 in MCF-7 cell line after EGF treatment (Figure 4D). Only the mutation of EBS3 sequence resulted in 30% decrease in *ZFP36 *promoter activation by Elk-VP16 (Figure 4B). Comparison of the sequence of TTP gene in different species revealed the presence of conservative elements in this region (Figure 4C). Importance of murine homologue of human EBS3 in serum responsiveness was already shown earlier [18]. We have confirmed the involvement of EGR-1 in the regulation of *ZFP36 *promoter by experiments with siRNA against EGR-1. The knockdown of EGR-1 in MCF-7 cells caused the lack of activation of *ZFP36 *promoter by EGF (Figure 4E). Taken together, we conclude that EGR-1 by possible interaction with EBS3 site can upregulate the activity of *ZFP36 *promoter.

**Figure 5:**
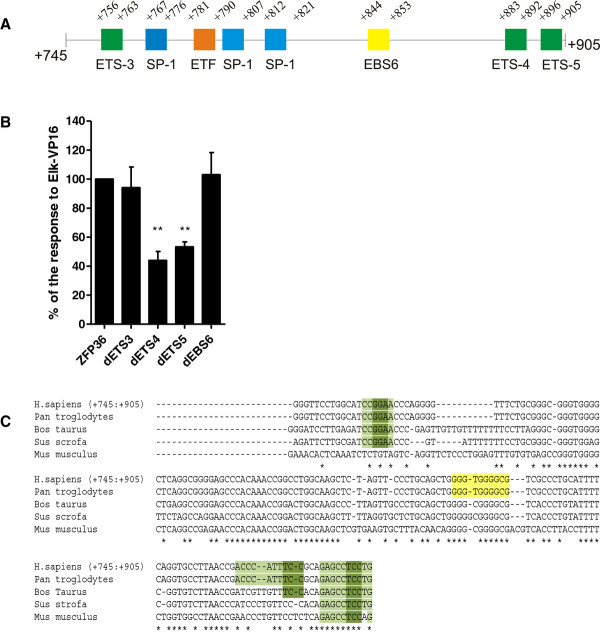
**Analysis of importance of sequences present in +745 to +905 bp of *ZFP36 *promoter fragment**. **(A) **The potential transcription factor binding sites identified in analyzed promoter fragment by Alibaba 2.1 and TRANSFAC 6.0 programs are shown. **(B) **MCF-7 cells were co-transfected with ZFP36 wild type or ZFP36 with mutation in ETS-3 (dETS-3), ETS-4 (dETS-4), ETS-5(dETS-5) or EBS6 (dEBS6) and 50 ng of pElk-VP16. 24 hrs after transfection luciferase activity was measured. **(C) **The multiple alignment of analyzed *ZFP36 *promoter fragment sequence homologues was performed by means of ClustalW2 program from the Emboss suite (GOP = 10, GEP = 5)

The region +744 to +905 bp contains three ETS sequences (ETS3, ETS4 and ETS5) which potentially can bind transcription factors from the ETS family and EBS6 sequence which can potentially interact with EGR-1 (Figure 5A). We have generated point-mutations of ETS3, ETS4 or EBS6 and deletion mutation of ETS5 in the full-length *ZFP36 *promoter (dETS3, dETS4, dETS5 or dEBS6). Despite high degree (90%) of conservation of EBS6 sequence among analyzed species (Figure 5B), its mutation did not influence the activation of *ZFP36 *promoter by Elk-VP16. Also mutation of ETS3 did not result in decrease of promoter activation. Mutations of ETS4 and ETS5 sequences leaded to about 50% reduction of Elk-VP16-induced up regulation of ZFP36 promoter activity (Figure 5B). These results suggest that ETS4 and ETS5 can participate in the regulation of *ZFP36 *promoter activity by ELK-1.

**Figure 6:**
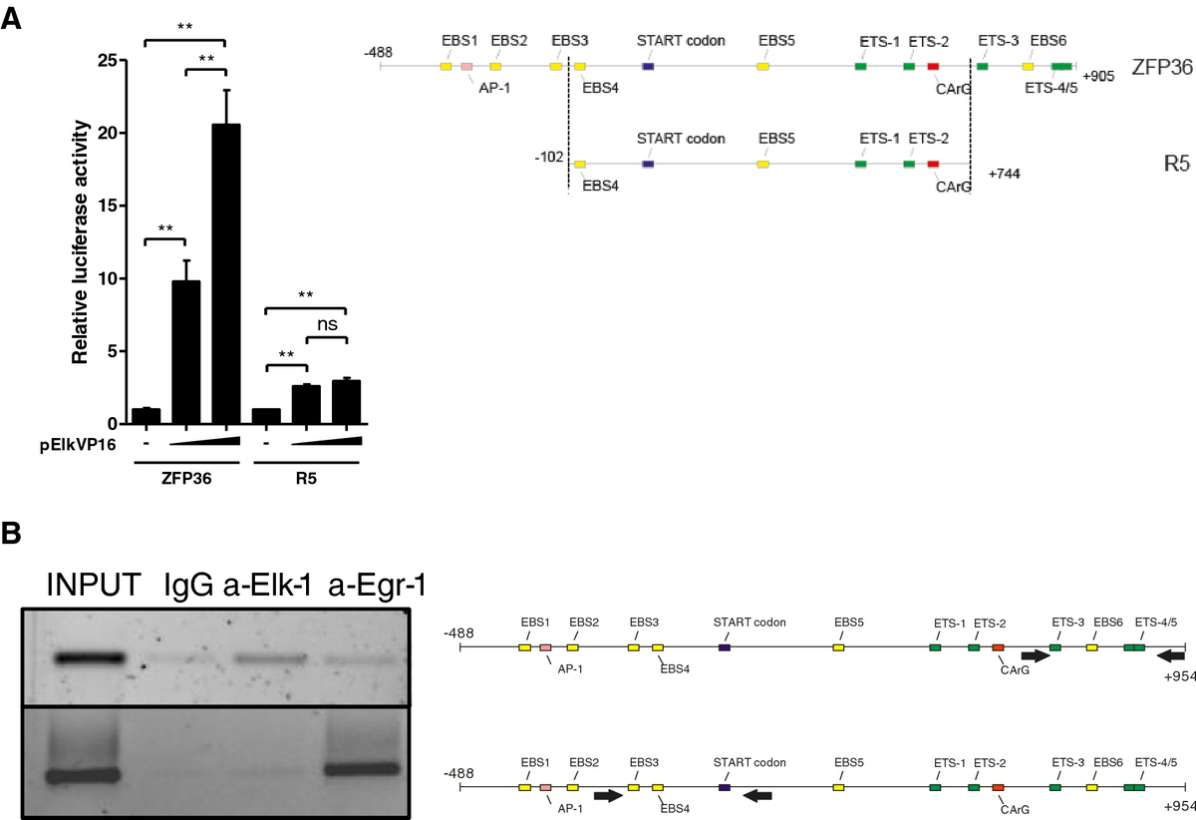
**Binding of ELK-1 and EGR-1 to *ZFP36 *promoter**. **(A) **MCF-7 cells were co-transfected with wild type ZFP36 and ZFP36 deletion mutant R5 (without -293 to -102 bp and +745 to +905 bp promoter fragment) and with or without pElk-VP16 (50 or 100 ng). 24 hrs after transfection luciferase activity was measured. Representative results from three independent experiments are shown and plotted as means ± SD (n = 3). (**P < 0,01). **(B) **Chromatin immunoprecipitation of transcription factors bound to *ZFP36 *promoter. Sonicated chromatin from MCF-7 cells grown in 10% FCS medium was immunoptrecipitated with either an ati-ELK-1 or anti-EGR-1 antibody or nonspecific IgG. PCR analysis of eluted DNA was performed using oligonucleotides specific for the distal region (upper panel) or proximal region (lower panel). 2% of input DNA is shown. The panels shown are inverted images of ethidium bromide-stained gels.

Since deletions of the regions containing EBS3 or ETS4/ETS5 (mutants F3 and R0 respectively) did not result in a loss of dose-dependent responsiveness to Elk-VP16 we decided to check whether deletion of both regions (mutant R5) will abolish this regulation. The results indicate that both investigated regions (-293 to -103 bp and +744 to +905 bp) are jointly needed for the regulation of *ZFP36*. Removing of both of them resulted in a loss of dose-dependent regulation of *ZFP36 *promoter by Elk-VP16 (Figure 6A).

To confirm the binding of EGR-1 to the sequence located -293 to -103 bp and the binding of ELK-1 to the sequence located +744 to +905 bp chromatin immunoprecipitation was performed. The lysates from MCF-7 cells were immunoprecipitated with anti-EGR-1, anti-ELK-1 or nonspecific antibody. By PCR with primers flanking the investigated sequences, the levels of immunoprecipitated promoter sequences was analyzed. We have observed increased level of -293 to -103 bp amplicon after immunoprecipitation with anti-EGR-1 antibody, in comparison to the level of template immunoprecipitated with anti-ELK-1 or nonspecific IgG (Figure 6B lower panel). When the primers flanking the region +744 to +905 bp were used, we have observed a higher amplification in samples immunoprecipitated with anti-ELK-1 antibody (Figure 6B upper panel). These results made us conclude that in vivo EGR-1 interacts with promoter sequence at the region -293 to -103 bp and ELK-1 interacts with the region +744 to +905 bp.

## Discussion

Our research focused on the mechanisms of TTP transcript induction by EGF in a cellular model of human breast cancer, using MCF-7 cell line. Our data indicate that the expression is under a stringent control of ERK1/2-dependent pathway. The activation of *ZFP36 *promoter by EGF is abolished when ERK1/2 pathway inhibitor, U0126, is present (Figure 1A, 2A). Dose-dependent regulation of investigated promoter (-488 to +905 bp) by constitutively active (Elk-VP16) and dominant-negative (Elk-EN) forms of ELK-1, suggests involvement of this transcription factor in the regulation of TTP transcription (Figure 2D). The dose-dependent activation of the investigated promoter by Elk-VP16 is lost when both regions of the promoter (-293 to -103 bp and +744 to +905 bp) are removed (Figure 6A). The region +744 to +905 bp contains two ETS sites (ETS4 and ETS5) important for the activation of the *ZFP36 *promoter by Elk-VP16. The sequence of ETS4 is GCGGAA, whereas the most frequent motif recognized by ELK-1 is CCGGAA. Such motif was characterized in *EGR-1, TR3, Pip92, MCL-1 and SRF *promoters [28-30]. However in other known ELK-1 target genes modifications of this canonical sequence are present. In c-*FOS *promoter ELK-1 binds to CAGGAT, in *nur77 *promoter to GAGGAA, in *MCPIP-1 *and *PAI-1 *to CAGGAA [27,29,31,32]. In the sequence of ETS5 (CAGGAG), the GGA core is preserved but the rest of the sequence is changed in respect to the canonical one (CCGGAA). The ETS4 and ETS5 sequences are located in close proximity and the disruption of any of them results in the similar effect (Figure 5B), which may suggest that ETS4 and ETS5 are in a functional relationship. A cooperation between proteins from ETS family was already described for promoters of stromelisine-1 and p53, where two Ets-1 proteins have to bind to the promoters to obtain their full activation. Recognition of ETS sequence through first Ets-1 molecule and its interaction with the second Ets-1 molecule results in conformational changes and formation of complex with DNA. This type of interaction between proteins and DNA enhances the affinity of the second transcription factor, even to the sequence which is not canonical [33,34]. It could not be excluded that ELK-1 forms a complex with other protein from ETS family on the *ZFP36 *promoter, especially that such complexes were described on other promoters [35-37]. For instance, the formation of heterodimer between Ets-1 and ELK-1 is a key step in the regulation of *DPP-III *gene expression. The C-terminal domain of ELK-1 and the N-terminal domain of Ets-1 are engaged in this interaction so that ETS sequences participating in this process have to be arranged in the orientation "head to tail". Such orientation of ETS4 and ETS5 is present in *ZFP36 *promoter which rises the possibility of such heterodimer formation on the investigated promoter.

EBS3 located at -111 to -103 bp turned out to be another sequence important for the regulation of human *ZFP36 *promoter by ELK-1 (Figure 4B). Murine EBS3 homologue was already shown to play a role in the regulation of *Zfp36 *promoter after serum stimulation [18].

ELK-1 activates *ZFP36 *promoter through EBS3 indirectly by stimulation of EGR-1 transcription which in turn binds to EBS3. Knockdown of EGR-1 in MCF-7 cells abrogates the activation of *ZFP36 *promoter by EGF (Figure 4E). Two other investigated EBS sequences (EBS2 located upstream from +1 and EBS6 located in the first intron) do not take part in the regulation of *ZFP36 *promoter by EGF. Also AP-1 binding site, despite activation of c-FOS by EGF in MCF-7 cell line (Figure 4D), is not important for the activation of TTP promoter by EGF. Lai et al [18] described the contribution of EBS3, AP2 and TPE1 (TTP promoter element 1) to the serum induction of murine *Zfp36 *promoter. Despite very high degree of conservation of all these elements in human and murine promoter we have detected only the importance of EBS3 in the regulation of human *ZFP36 *promoter by EGF.

We hypothesize that the regions containing EBS3 and ETS4/ETS5 are equally important for the stimulation of TTP expression by EGF. Removing of both regions resulted in a complete loss of dose-dependent regulation of the promoter by Elk-VP16 (Figure 6A) and point mutations of any of these sites abrogated the EGF-dependent promoter activation (data not shown). Elimination of EGR-1 from the cells causes the same effect (Figure 4E). Neither EBS3 nor ETS4/5 site is sufficient enough to drive the activation of *ZFP36 *promoter alone. The binding of ELK-1 and EGR-1 to *ZFP36 *promoter detected by means of chromatin immunoprecipitation confirmed involvement of these transcription factors in the regulation of TTP expression.

## Conclusions

**Figure 7:**
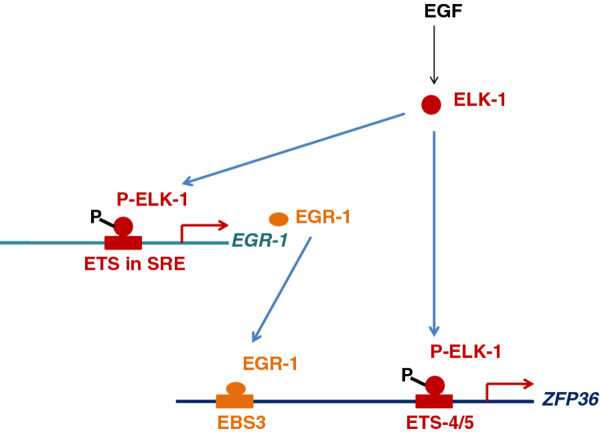
**Schematic diagram of regulation of the *ZFP36 *promoter by EGF**.

EGF regulates *ZFP36 *expression through activation of transcription factor ELK-1. ELK-1 binds directly to the *ZFP36 *promoter through the sequences localized at + 883 to +905 bp. ELK-1 induces also the expression of another transcription factor EGR-1 which as well binds to the *ZFP36 *promoter to the sequence at -111 to -103 bp (Figure 7). TTP was shown to negatively modulate a number of factors connected with mammary gland tumor progression. Among them IL-6, COX-2, c-FOS, urokinase, urokinase receptor, metalloproteinase-1 can be pointed out and notably, all of them are down-modulated at their mRNA level by tristetraprolin [6,38-40]. Our results demonstrate that the expression of *ZFP36 *is stimulated by EGF. The results show complex influence of EGF on the development of breast cancer. EGF is well-known as a factor which promotes tumor growth and survival. This growth factor is able to induce heterodimerization between HER-2/Neu (c-ErbB2) and its exclusive receptor c-ErbB1. Elevated levels of c-ErbB1 and HER-2 in breast cancer is correlated with high disease recurrence rates and decreased patient survival [41]. Upregulation of TTP expression by EGF described in this paper reveals unexpected influence of EGF on breast cancer development. TTP expression is diminished in many cancers and overexpression of TTP in tumors delayed tumor growth and vascularization [42-44]. Thus induction of TTP expression by EGF can be classified as a anti-tumor activity of this growth factor.

## Materials and methods

### Cell culture

Adenocarcinoma cell line MCF-7 (ATCC No HTB-22), human embryonic kidney epithelium cell line HEK293 (ATCC No CRL-1573), human epithelium cell line HeLa (ATCC No CCL-2) and human hepatoma cell line (HepG2 No CRL-10741) were cultured at 37°C and 5% CO_2_. MCF-7 cells in Minimal Eagle's Medium (MEM) (Sigma) supplemented with 10% FBS and bovine insuline (10 ug/ml), HEK293, HeLa and HepG2 in Dulbecco's modified Eagle's Minimum Essential Medium (DMEM, Gibco) with 1 g/L D-glucose supplemented with 10% FBS.

### Reagents and cell stimulation

Cells were stimulated with EGF (20 ng/ml) or PMA (100 ng/ml) (R&D). When applied, the inhibitor of MEK1/2, U0126 (10 μM) (Calbiochem) was added to the medium 30 min prior stimulation. Pre-designed oligonucleotide Silencer siRNA targeted to EGR-1 (Cat#16810) and control one (Cat# 4611) were supplied by Ambion.

### RNA preparation and northern blot analysis

Total RNA isolation and northern blot analysis was performed as described previously [28].

### Plasmid constructs

pEF1/Myc-His/lacZ is a control vector containing the gene for β-galactosidase (Invitrogen). pElk-VP16, pElk-EN were described before [28]. pZFP36 containing human *ZFP36 *promoter fragment (-488 to +905 bp) was generated by two step PCR, using total DNA isolated from MCF-7 cells. The first round PCR was carried out with the primer forward: 5' GTCTTCCCTCCCTTCCTCAC 3' and reverse 5' GTCAGGGCTCAGCGACAG 3'. Then the second round was performed with nested primers introducing *SacI and BglII *restriction sites: forward: 5' TCGAGCTCTTCCTCACCCTGTCTATC 3' and reverse: 5' TCAGATCTCAGGAGGCTCTGCGGAAATG 3'. The introduced restriction sites were used during cloning of PCR product to pGL2-Basic reporter vector (Promega). The set of deletion mutants was prepared using pZFP36 as a template. The following forward primers with restriction site for SacI or NheI were used in PCR reactions:

F1: 5' GCTAGCCAGTTTCCTTCTACAAGCCTCAG 3'

F3, R5: 5' GCTAGCCGCGTCCGGGAAG 3'

F5: 5' GAGCTCGCGTCCGGGAAGC 3'

F6: 5' GAGCTCGGCCCCGGCCCCGG 3

The set of promoter constructs lacking different 3'-terminal sequences were generated with the following reverse primers:

R01: 5' TCAGATCTGGAACTAGAGCTTGCCAG 3'

R0, R5: 5' TCAGATCTAGAGTTGGAGGTTCTGAG 3'

R1: 5' CTTAAGCACGCGTCGGGATCTC 3'

R2: 5' CTTAAGTTTGAGCGAAGAGCCGGGTG 3'

R4, F6, F5: 5' GCTAGCCTCGTAGATGGCAGTCAG 3'

The constructs containing mutations in selected transcription factors' binding sites were generated using QuikChange XL Site-Directed Mutagenesis kit (Stratagene) according to the manufacturer's procedure. The sequences were changed as follows:

EBS2: CCCCCACCCC to CCCCCAgCtg

EBS3: CCGGGGGCG to CCatGGGCG

AP-1: CAAGCCTCAG to CAAGCCatgG

ETS3: ATCCGGAA to tgCaGccA

ETS4: ACCCATTTCC to ACCCATggCC

EBS6: GGGTGGGGCG to GGGTaccGCG

The point mutant ETS5 was created by means of deletion mutation after a PCR reaction performed with a reverse primer 5' TCAGATCTTGCGGAAATGGGTCGGT 3'.

### Reporter gene assay

Transient transfection experiments were carried out using Lipofectamine 2000 reagent (Invitrogen) in 12-well plate. Total amount of 1.6 μg of DNA per each well was used, including 0.4 μg of reporter vectors with *ZFP36 *promoter fragments and 10 ng of pEF1/Myc-His/lacZ. For some experiments indicated amounts of pElk-VP16 or pElk-EN were used. The amount of DNA per well was equalized using mock DNA (pcDNA3). Luciferase assays were carried out using the dual light reporter gene assay system (Tropix) according to the manufacturer's procedure. Luciferase activity was measured 24 hrs after transfection or at indicated time point after stimulation. β-galactosidase activity was measured to normalize the efficiency of transfection. All experiments were repeated at least three times in duplicates.

### Western blot

Western blot was carried out using Immobilon Western chemiluminescent HRP substrate (Millipore) and anti-phospho ERK1/2 (Cell Signaling), anti-ELK-1 (Santa Cruz), anti- phospho p38 (Cell Signaling), anti-phospho JNK (Abcam), anti-c-FOS (Santa Cruz), anti-EGR-1 (Abcam), anti-GAPDH (Abcam), anti-phospho Ser383 ELK-1 (Santa Cruz), anti-ERK, anti-p38, anti-JNK (Cell Signalling).

### siRNA/DNA co-transfection

Was carried out using Lipofectamine 2000 reagent (Invitrogen) in 12-well plate. Total amount of 600 ng plasmid DNA and 50 pmol siRNA oligonucleotides per each well was used.

### Chromatin immunoprecipitation assay

Chromatin immunoprecipitation was carried out as described before [28] using anti-ELK-1, anti-EGR-1 (Santa Cruz) and nonspecific IgG (Upstate). Following primers forward: ACCTCCAACTCTGGGTTCCT and reverse: GACTCAGTCCCTCCATGGTC for fragment containing ETS4/5 sites and forward: CGGAAGGGAACCAGTCCAG and reverse: AGAGTGGGAGCGCTGAAGT for fragment containing EBS3 binding site were used.

## Authors' contributions

MF, PT, AB, AS constructs generation, reporter gene assays. AS qPCR. PT, JK, MW, KS western blots. AK chromatin immunoprecipitation, conception and interpretation of data. MF, PT, AK writing of manuscript. All authors drafted, read and approved the manuscript.
